# Infant-specific gaze patterns in response to radial optic flow

**DOI:** 10.1038/srep34734

**Published:** 2016-10-06

**Authors:** Nobu Shirai, Tomoko Imura

**Affiliations:** 1 Department of Psychology, Faculty of Humanities, Niigata University 2-8050 Ikarashi Nishi-Ku Niigata, 950-2181, Japan; 2 Department of Information Systems, Faculty of Information Culture, Niigata University of International and Information Studies, 3-1-1, Mizukino, Nishi-ku, Niigata, 950-2292, Japan

## Abstract

The focus of a radial optic flow is a valid visual cue used to perceive and control the heading direction of animals. Gaze patterns in response to the focus of radial optic flow were measured in human infants (*N* = 100, 4–18 months) and in adults (*N* = 20) using an eye-tracking technique. Overall, although the adults showed an advantage in detecting the focus of an expansion flow (representing forward locomotion) against that of a contraction flow (representing backward locomotion), infants younger than 1 year showed an advantage in detecting the focus of a contraction flow. Infants aged between 13 and 18 months showed no significant advantage in detecting the focus in either the expansion or in the contraction flow. The uniqueness of the gaze patterns in response to the focus of radial optic flow in infants shows that the visual information necessary to perceive heading direction potentially differs between younger and mature individuals.

Moving around the environment is fundamental to the nature of all animals. Optic flow, the motion pattern that appears in the visual field during self-motion, contributes to the perception and control of the direction of self-motion in the environment. For instance, when an individual moves forward, a radially expanding optic flow typically appears on the individual’s retina. In such a situation, the position of the focus of the radial optic flow in the visual field is always consistent with the heading direction of the individual at any given moment^1^. Despite some controversy^2^,^3^, it has been empirically demonstrated that the focus of radial optic flow can serve as a cue for perceiving and controlling the direction of self-motion^4^,^5^,^6^ (see also a series of discussions by Lappe *et al*. and Harris & Rogers^7^,^8^,^9^). Moreover, we seem to have a special ability to detect the focus of radial optic flow relevant to our locomotor action; even naive adults tend to focus their gaze toward the focus of radial optic flow during observation of radial flow stimuli. This tendency is more remarkable when observing an expansion flow, which corresponds to forward locomotion, than a contraction flow, which corresponds to backward locomotion^10^.

Although numerous studies^11^,^12^,^13^,^14^,^15^,^16^,^17^ have shown that significant radial optic flow sensitivity develops in the first few months of life, only a few studies have empirically investigated how this special ability, the detection of the focus of radial optic flow, changes with development. Gilmore and colleagues^18^,^19^ have shown that a precursor to this ability appears very early in life; even 3–5-month-old infants who had no ability to execute voluntary locomotion could discriminate between radial flows that had different focus positions. However, the infants’ discrimination threshold for the position of the focus of radial optic flow (>20 deg) was much higher than that of adults (<2 deg), and there was no significant improvement in this threshold throughout early infancy. Moreover, because the analysis combined the infants’ responses to both expansion and contraction flows, it is difficult to determine whether the infants also had an advantage in detecting the focus of an expansion flow against that of a contraction flow. Thus, how and when the gaze patterns in response to the focus of radial optic flow mature during development remain open questions.

Here, we report on how the gaze patterns in response to the focus of radial optic flow develop during infancy. Naive adults are more sensitive to detecting the focus of an expansion than a contraction, as shown by a previous study^10^. Using an eye-tracking technique, we found that infants younger than 12 months have the opposite tendency: the younger infants were more sensitive to the focus of a contraction than to that of an expansion. This advantage in the detection of the focus of a contraction disappeared in older infants (13–18-months), although they did not show an advantage in detecting the focus of an expansion, as would be expected in adults. Additionally, the infants tended to see relatively peripheral areas of radial flows, whereas the adults tended to concentrate their gaze on areas near the focus of radial optic flow. These results suggest that, although gaze patterns in response to the focus of radial optic flow change dramatically during infancy, they remain qualitatively and quantitatively different from those of adults, even at later stages of infancy.

## Results

**Figure 1:**
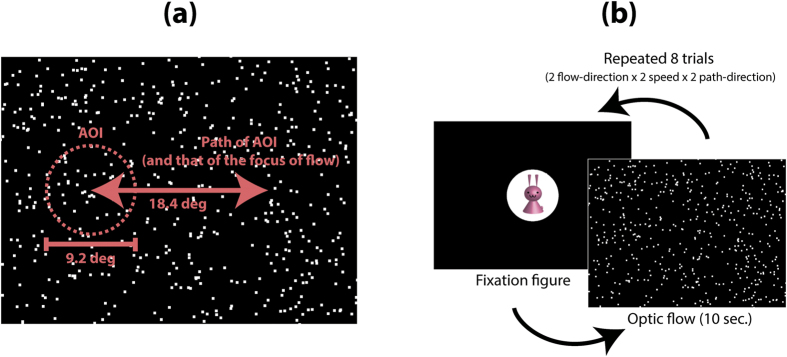
(**a**) A schematic diagram of the motion paths of the area of interest (AOI) and the focus of the radial flow pattern. (**b**) A flowchart of the experimental procedure.

One hundred infants (20 infants per age group: 4–6 months, 7–9 months, 10–12 months, 13–15 months, and 16–18 months) and 20 naïve adults participated in the experiment. In each experimental trial, either a radial expansion or a radial contraction flow (35.3 degree × 26.4 degree), composed of 500 moving dots appeared on a computer screen as a visual stimulus. The focus of radial optic flow moved horizontally back and forth (temporal frequency = 0.2 Hz) between the right and the left sides of the screen (18.4 degrees). Each participant engaged in eight experimental trials (“expansion/contraction” × “high/low dot speed” × 2 repetitions). Participants’ gaze patterns during each trial were recorded by an eye tracker, and a circular area of interest (AOI; subtended 9.2 degrees) was set around the focus of radial optic flow (Fig. 1). Two measurements, the latency of the first gaze at the AOI and the total time looking at the AOI in each trial, were used as dependent variables.

### Looking time

**Figure 2:**
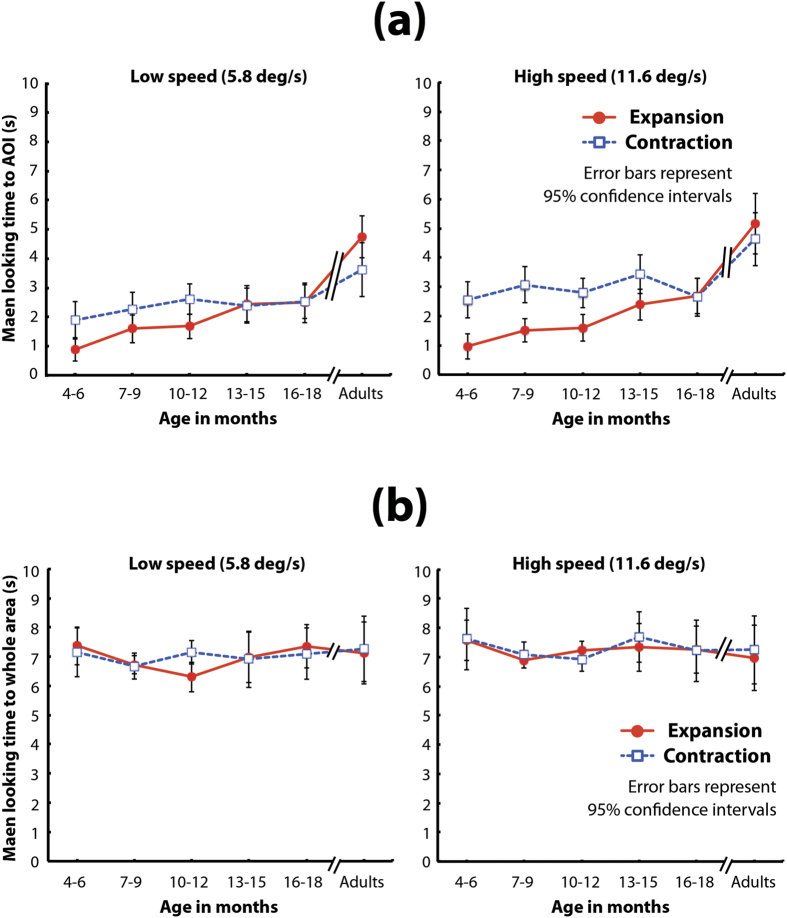
(**a**) Mean time looking at the AOI of the flow patterns. In each graph, the vertical axis represents mean time looking at the AOI, and the horizontal axis represents age group. Solid lines with filled circles indicate mean time looking at expansion flow patterns, and broken lines with open squares indicate mean time looking at contraction flow patterns. The left and right graphs show the results under the low-speed condition and under the high-speed condition, respectively. Error bars indicate 95% confidence intervals. (**b**) Mean time looking at the whole area of the flow patterns. In each graph, the vertical axis represents mean time looking at a whole pattern, and the horizontal axis represents age group. Solid lines with filled circles indicate mean time looking at expansion flow patterns, and broken lines with open squares indicate mean time looking at contraction flow patterns. The left and right graphs show the results under the low-speed condition and under the high-speed condition, respectively. Error bars indicate 95% confidence intervals.

First, we analyzed the results for time spent looking (Fig. 2a) with a mixed-design three-way analysis of variance (*ANOVA*: age [6] × flow direction [2] × dot speed [2]). The *ANOVA* revealed that the main effects of age, flow direction, and dot speed were significant (*F*(5,114) = 19.98, *p* < 0.001, *η*_*p*_^2^ = 0.47; *F*(1,114) = 26.26, *p* < 0.001*, η*_*p*_^2^ = 0.18; and *F*(1,114) = 15.06, *p* < 0.001, *η*_*p*_^2^ = 0.12; respectively). The interaction between age and flow direction was significant (*F*(5,114) = 10.52, *p* < 0.001*, η*_*p*_^2^ = 0.32). The simple main effect of age on time spent looking at the expansion flow was significant (*F*(5,228) = 29.27, *p* < 0.001*, η*_*p*_^2^ = 0.39). A multiple comparison by two-tailed Bonferroni-corrected *t-*tests revealed that the time the 4–6-month-olds spent looking at the AOI in the expansion flow was significantly shorter than that spent by the 13–15-month-olds and the 16–18-month-olds (*ps* < 0.05). Additionally, the time spent looking by the adults was significantly longer than that spent by all the other age groups (*ps* < 0.05). The simple main effect of age on the time spent looking at contraction flows was also significant (*F*(5,228) = 6.31, *p* < 0.001*, η*_*p*_^2^ = 0.12). A multiple comparison by two-tailed Bonferroni-corrected t-tests revealed that the time adults spent looking at the AOI in contraction flows was significantly longer than that spent by all other age groups (*ps* < 0.05). Moreover, the simple main effects of flow direction on the 4–6- and 7–9-month-old infants and on the adults were significant (*F*(1,114) = 26.52, *p* < 0.001, *η*_*p*_^2^ = 0.19; *F*(1,114) = 19.71, *p* < 0.001*, η*_*p*_^2^ = 0.15; and *F*(1,114) = 10.75, *p* = 0.002*, η*_*p*_^2^ = 0.09; respectively); it was marginally significant on the 10–12-month-olds (*F*(1,114) = 3.89, *p* = 0.051*, η*_*p*_^2^ = 0.13). The interaction between flow direction and dot speed was also significant (*F*(1,114) = 11.35, p < 0.001*, η*_*p*_^2^ = 0.03). The simple main effect of flow direction under the high-speed condition was significant (*F*(1,228) = 25.82, p < 0.001*, η*_*p*_^2^ = 0.10). Moreover, the simple main effects of dot speed on time spent looking at the expansion flows was marginally significant (*F*(1,228) = 3.38, p = 0.067*, η*_*p*_^2^ = 0.01), whereas dot speed had a significant effect on the time spent looking at the contraction flows (*F*(1,228) = 36.50, p < 0.001*, η*_*p*_^2^ = 0.14). The interactions between age and dot speed and among the three factors were not significant (*F*(5,114) = 1.09, *p* = 0.368*, η*_*p*_^2^ = 0.05 and *F*(5,114) = 1.02, *p* = 0.412*, η*_*p*_^2^ = 0.04, respectively).

Interestingly, adults and infants differed with regard to the direction of the expansion/contraction flow for which they showed an advantage. Results from the adults were mostly consistent with their known advantage for detecting the focus of an expansion flow^10^, as the adults looked significantly longer at the focus of an expansion flow than at that of a contraction flow. In contrast, the 4–6- and 7–9-month-old infants looked significantly longer at the focus of a contraction flow than at that of an expansion flow. Additionally, the advantage for contraction flows was marginally significant in infants aged 10–12 months and disappeared in the 13–15- and 15–18-month-olds.

**Figure 3:**
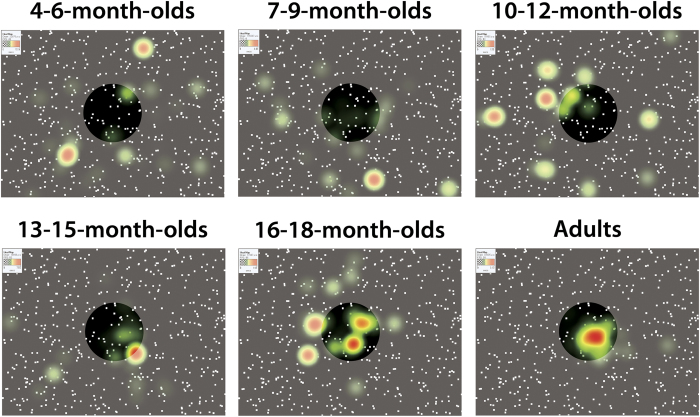
Snapshots taken from movies showing heat maps of gaze patterns in response to an expansion flow pattern with low dot speed (5.8 deg/s) in all age groups (*N* = 20 for each group). In each movie, the focus of radial optic flow (and thus AOI) was moving “from left to right to left” through the presentation. Snapshots were taken 7.5 seconds from the onset of the expansion flow and, thus, at the moment the focus of radial optic flow appeared at the center of the presentation field. The AOI is shown as a highlighted circle in each snapshot. Note that the highlighted circles are virtual ones and did not appear during the experiment. The original movies are available at “ https://nyu.databrary.org/volume/252”.

Another important finding is that the time spent looking at the AOI significantly increased with age. The youngest age groups spent a shorter amount of time looking at the AOI than did the other age groups, and the adults spent a longer amount of time looking at the AOI than did the other age groups. The difference between infants and adults in time spent looking at the AOI should stem from a specific trend in the gaze patterns in response to the focus of radial optic flow rather than from the looking behaviors elicited by the whole optic flow patterns. We found that there was no significant difference among age groups in the time spent looking at the whole area of each visual stimulus (Fig. 2b). We set another AOI (AOIW; for details, see the ‘Experimental conditions and data analyses’ section under ‘Method’), which fully covered the entire area of the presentation field, and analyzed the time looking at AOIW for each visual stimulus. A mixed-design three-way *ANOVA* (age [6] × flow direction [2] × dot-speed [2]) revealed that the main effect of only dot speed was marginally significant (*F*(1,114) = 3.61, *p* = 0.060, *η*_*p*_^2^ = 0.03). Other main effects and interactions were not significant (age: *F*(5,114) = 0.40, *p* = 0.846, *η*_*p*_^2^ = 0.02; flow direction: *F*(1,114) = 0.33, *p* = 0.565, *η*_*p*_^2^ = 0.00; age × flow direction: *F*(5,114) = 0.29, *p* = 0.916, *η*_*p*_^2^ = 0.01; age × speed: *F*(5,114) = 0.58, *p* = 0.717, *η*_*p*_^2^ = 0.02; flow direction × speed: *F*(1,114) = 0.01, *p* = 0.943, *η*_*p*_^2^ = 0.00; age × flow direction × speed: *F*(5,114) = 0.98, *p* = 0.434, *η*_*p*_^2^ = 0.04). The lack of the significance of the main effects and the interactions involving age means that infants and adults spent almost the same amount of time looking at the whole visual displays. In other words, although infants and adults spent almost the same time looking at the whole flow pattern, the infants spent more time looking at relatively peripheral areas of the whole flow pattern, whereas the adults spent more time looking at a relatively central area near the focus of radial optic flow. Indeed, such differences in looking patterns between infants and adults are remarkable when their gaze patterns are plotted as spatiotemporal heat maps (Fig. 3, see also supplemental movies) Taken together, the results for looking time indicate that the gaze patterns in response to the focus of radial optic flow change drastically through infancy, but the gaze patterns of infants remain different from those of adults even in the later stage of infancy.

### Latency

**Figure 4:**
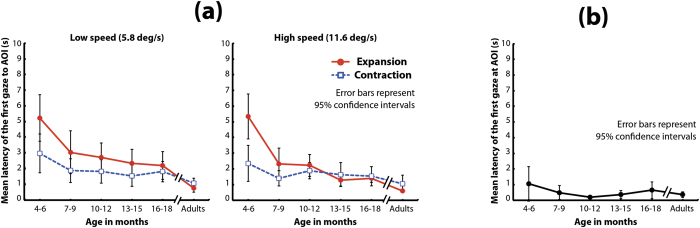
(**a**) Mean latency of the first gaze toward the AOI of the flow patterns. In each graph, the vertical axis represents the mean latency to the AOI, and the horizontal axis represents age group. Solid lines with filled circles indicate the mean latency for expansion flow patterns, and broken lines with open squares indicate the mean latency for contraction flows. The left and right graphs show the results under the low-speed condition and under the high-speed condition, respectively. Error bars indicate 95% confidence intervals. (**b**) Mean latency of the first gaze at the AOI of the benchmark patterns. The vertical bar represents the mean latency to the AOI, and the horizontal bar represents age group. Error bars indicate 95% confidence intervals.

Next, we analyzed the latency of the first gaze toward the AOI (Fig. 4a). A mixed-design three-way *ANOVA* (age [6] × flow direction [2] × dot speed [2]) revealed that the main effects of age, flow direction, and dot speed were significant (*F*(5,114) = 15.16, *p* < 0.001*, η*_*p*_^2^ = 0.40; *F*(1,114) = 22.93, *p* < 0.001*, η*_*p*_^2^ = 0.17; and *F*(1,114) = 5.27, *p* = 0.024*, η*_*p*_^2^ = 0.04, respectively). The interaction between age and flow direction was also significant (*F*(5,114) = 8.56, p < 0.001*, η*_*p*_^2^ = 0.27). The simple main effect of age on expansion was significant (*F*(5,228) = 23.50, *p* < 0.001*, η*_*p*_^2^ = 0.34). A multiple comparison by two-tailed Bonferroni-corrected t-tests revealed that the latency of the first gaze toward the AOI of the expansion flow by 4–6-month-olds was significantly longer than those of all other age groups (*ps* < 0.05). Additionally, the latency in adults was significantly shorter than those in the 4–6-, 7–9-, and 10–12-month-olds (*ps* < 0.05). The simple main effect of age on the latency in response to the contraction flow was also significant (*F*(5,228) = 2.64, *p* = 0.024*, η*_*p*_^2^ = 0.05). A multiple comparison by two-tailed Bonferroni-corrected t-tests revealed that the latency for gazing at the AOI of the contraction flow in 4–6-month-olds was significantly longer than it was in adults (*p* < 0.05). Moreover, the simple main effects of flow direction among those aged 4–6 and 7–9 months were significant: (*F*(1,114) = 52.81, *p* < 0.001, *η*_*p*_^2^ = 0.32 and *F*(1,114) = 8.39, *p* = 0.005*, η*_*p*_^2^ = 0.07), respectively. It was marginally significant among those aged 10–12 months: (*F*(1,114) = 2.87, *p* = 0.093*, η*_*p*_^2^ = 0.02). The interactions between age and dot speed, between flow direction and dot speed, and among the three factors were not significant: (*F*(5,114) = 0.26, *p* = 0.932*, η*_*p*_^2^ = 0.01; *F*(1,114) = 1.33, *p* = 0.251*, η*_*p*_^2^ = 0.01; and *F*(5,114) = 0.89, *p* = 0.494*, η*_*p*_^2^ = 0.04; respectively).

The main finding in the analysis of latency was that the latency for detecting the focus of the contraction flow was shorter than that it was for detecting the latency of the expansion flow in infants aged 4–6 and 7–9 months. This difference in the latencies between expansion and contraction flows changed modestly in the 10–12-month-olds and was not present in the 13–15- and 16–18-month-olds or in the adults. The advantage for detecting the focus for contraction versus expansion flows, observed only in the younger infants, seems to be consistent with the results of the analyses of looking times: the mean time spent looking at the focus of contraction was greater than that spent looking at the focus of expansion in the same age groups.

Another finding is that the infants tended to show longer latency for gazing at the AOI than did the adults. For instance, the infants aged 4–6, 7–9, and 10–12 months showed longer latency to detect the focus of the expansion flow than did the adults. Additionally, the youngest infants showed significantly longer latency than did the adults for detecting the focus of contraction flows. These results suggest that the gaze patterns of younger infants in response to the focus of radial optic flow were less sluggish than were those of adults, particularly for expansion flows. One may speculate that the longer latency in the young infants might be due to the infants’ immature eye movement rather than to issues related to the detection of the focus of radial optic flow. However, we found no significant difference between infants and adults in the latency for detecting a benchmark stimulus (a white small square moved in exactly the same motion path as the focus of radial optic flow; see the Method section for details) (one-way *ANOVA*: *F*(5,114) = 1.31, *p* = 0.266*, η*_*p*_^2^ = 0.05; see also Fig. 4b). Hence, the longer latency in the young infants might reflect specific gaze patterns in response to the focus of radial optic flow rather than the immature eye movement characteristic of infancy.

## Discussion

Overall, the mean time spent looking at the focus of radial optic flow was shorter in the infant groups than in the adults, and the mean latency to the first gaze at the focus of radial optic flow was longer in the infant groups than in the adults. These results indicate that the gaze behaviors in response to the focus of radial optic flow quantitatively differed in infancy and adulthood. These results are consistent with those of previous studies reporting very high thresholds in young infants for discriminating between optic flow patterns with different positions in the focus of radial optic flow^18^,^19^. On the other hand, the current study offers new findings about the development of the gaze patterns in response to the focus of radial optic flow; specifically, there are drastic developmental changes in the gaze patterns toward the focus of radial optic flow across infancy.

The most unique finding of the current study is that the youngest infants, in particular those aged less than 1 year, had qualitatively different gaze patterns in response to the focus of radial optic flow compared with the adults; the young infants showed an advantage for detecting the focus of a contraction flow, whereas the adults showed an advantage for detecting the focus of an expansion flow. Because an expansion flow is a strong visual cue for perceiving heading direction, the benefits of an advantage for detecting the focus of an expansion flow are clear. However, it is difficult to explain the benefits of having an advantage for detecting the focus of a contraction flow. There are several possible explanations for the “contraction advantage” in younger infants, which we discuss below.

One possibility is that younger infants’ avoidance responses to an approaching object might have affected the current results. A radial flow pattern can be a visual cue to perceive an approaching object, and young infants show avoidance responses, such as head rotation and blinking, in response to a large-field expansion flow under optimum experimental settings^20^,^21^,^22^,^23^,^24^. It is plausible that the young infants avoided looking at expansion flows; thus, their looking behaviors in response to the focus of radial optic flow under the expansion condition decreased. However, such a possibility is ruled out for the results of the analysis of the time spent looking at the whole visual stimuli. As shown in the Results section, there was no significant age difference in the time spent looking at the whole area of the expansion versus the contraction flow. This means that even the younger infants looked at the expansion and the contraction flows equally; thus, there is no evidence for the younger infants’ avoidance of looking at the expansion flows.

Alternatively, the infants may have been responding to differences in the lower-level visual properties of the expansion and contraction flow patterns. For instance, a dynamic random dot pattern generally has a non-uniform distribution of dot density across the presentation field. Because moving dots tend to accrete around the edge in the direction of the dots, dot density tends to be higher near the edge in the direction of the dots. Such a difference in visual properties, which is not directly related to the direction of radial expansion/contraction, may have unexpected effects on infants’ gaze patterns. However, our visual stimuli controlled for this kind of artifact. All our contraction flow movies shared common animation frames with the corresponding expansion flow movies; for instance, a contraction flow with a high dot speed in which the focus moved from left to right to left was generated by presenting exactly the same set of animation frames as those for an expansion flow in which the focus moved from left to right to left with high dot speed in reverse order (see also the ‘Stimulus’ section under ‘Method’). Thus, in the current study, no differences were observed in any visual properties other than the direction of motion between the expansion and contraction flow movies. On the other hand, it may be necessary to consider the effect of an apparent difference between the expansion and contraction flows on young infants’ gaze behaviors. Generally, human adults perceive that moving patterns shift in an illusory way toward the direction of the motion of such patterns^25^. Thus, although there has been no empirical evidence that young infants perceive the illusory position shift of moving objects, the positions of dots might appear to shift toward the central/peripheral area in a contraction/expansion flow in the current experiment. Such a potential difference in the apparent dot distribution in the expansion and contraction flows might result in specific gaze patterns directed at the radial flow stimuli; that is, the younger infants might prefer an apparently denser/brighter area in a radial flow pattern.

Another explanation for the observed “contraction advantage” is that the young infants may have been responding to a local subset of dots within the overall global flow pattern. It is known that young infants, even newborns, shift their gaze in the direction of a moving visual pattern^26^,^27^. It is possible that local motion within a flow pattern attracted the young infants’ gaze toward the direction of local motion. It should be noted that when an observer focuses his/her attention on a relatively local area of a whole contraction (or expansion) flow pattern, the direction of the local subset of moving dots should be perceived as translational motion headed toward (or away from) the focus of a contraction (or an expansion). If young infants’ attention were easily captured by relatively local translational motion rather than by the focus of radial optic flow, as defined by the global structure of the whole flow pattern, then the infants would show an apparent contraction advantage in their gaze patterns. Because the focus of radial optic flow seems to be ecologically less important as visual information for young infants, their oculomotor system may not need to be as sensitive to the focus of radial optic flow. Raudies *et al.*^28^ reported marked differences in optic flow during forward locomotion by 9.5-month-old infants versus adults in normal daily situations. They recorded typical views of dynamic visual scenes experienced by infants and their mothers using head-mounted cameras and wearable eye-tracking glasses in situations in which the mothers walked forward while holding their babies in a forward-facing carrier. They found that the optic flow experienced by young infants contained a greater number of translational components and fewer radial components than did that experienced by adults. Moreover, the position of the focus of radial optic flow was higher in the infants’ visual field than in the adults’ visual field. Their results imply that young infants may experience a less radially structured optic flow during locomotion in their daily lives. Moreover, the results of another study (Raudies & Gilmore)^29^, obtained under an experimental setting similar to the one employed by Raudies *et al.*^28^, suggest that 9-month-old infants who are carried experience optic flow that consisted of higher-speed and more vertically-directed motion components relative to their mothers during forward locomotion. Presumably, a young infant’s oculomotor system is more sensitive to the translational motion in the optic flow patterns typically seen by, and thus familiar to, young infants, than it is to radial motion.

**Figure 5:**
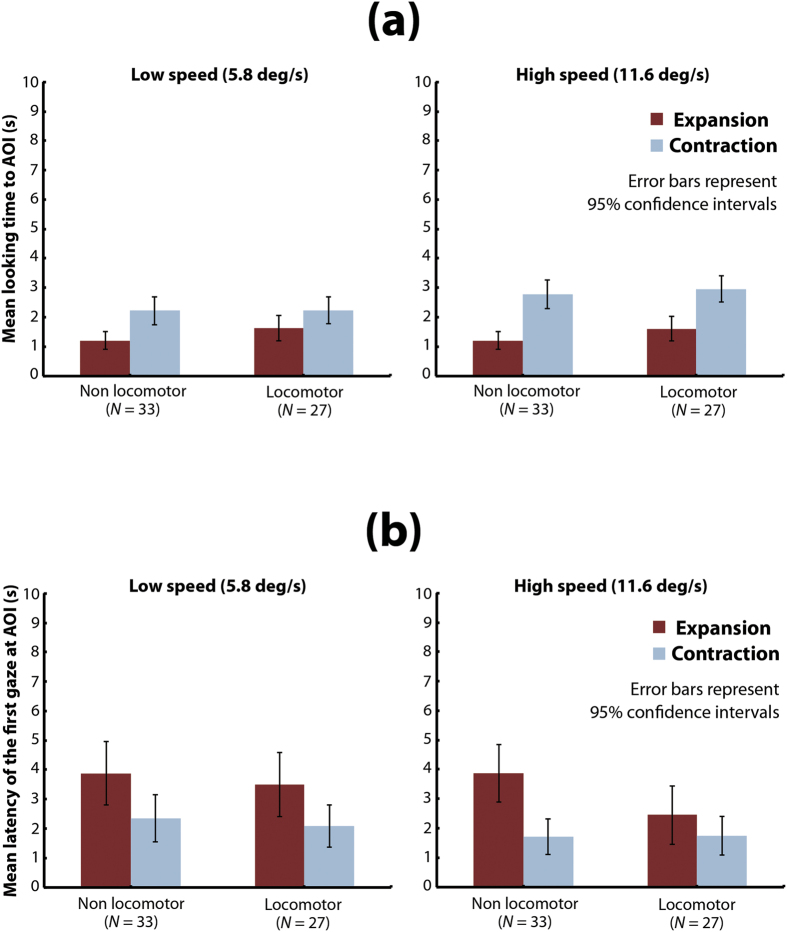
Analysis of the effect of locomotor experience on gaze patterns in response to the AOI in younger (≦12 months) infants. (**a**) Mean time looking at the AOI of the flow patterns. In each graph, the vertical axis represents mean time looking at the AOI, and the horizontal axis represents the infants’ locomotor state. Dark bars indicate mean time looking for expansion flow patterns, and pale bars indicate mean time looking for contraction flow patterns. The left and right graphs show results under the low-speed condition and under the high-speed condition, respectively. Error bars indicate 95% confidence intervals. (**b**) Mean latency of the first gaze at the AOI of the flow patterns. In each graph, the vertical axis represents the mean latency to the AOI, and the horizontal axis represents the infants’ locomotor state. Dark bars indicate the mean latency for expansion flow patterns, and pale bars indicate the mean latency for contraction flow patterns. The left and right graphs show results under the low-speed condition and under the high-speed condition, respectively. Error bars indicate 95% confidence intervals.

Another important finding in the current results is the developmental change in the contraction advantage: the contraction advantage disappeared in later infancy (13–15 and 16–18 months). Several studies have shown extensive interactions between the development of optic flow perception and that of motor responses (e.g., voluntary locomotion) in young infants^30^,^31^,^32^. For instance, Shirai and Imura^32^ reported that visual preference for optic flows, particularly radial contraction flows, drastically changes around the developmental onset of voluntary locomotion in infants, which occurs by 12 months. Thus, it is reasonable to infer that even young infants would show no or reduced contraction bias if they had engaged in sufficient voluntary locomotion. To examine this, we divided the infants aged 4 to 12 months who had participated in the main experiment into two groups (non-locomotor infants: *N* = 33, mean age = 192.1 days, *SD* = 66.2; locomotor infants: *N* = 27, mean age = 295.7 days, *SD* = 45.6) based on the results of a behavioral test for locomotor ability (identical to that used by Shirai and Imura 2014: for details, see Method section). We then compared the mean looking time and mean latency between the two groups (Fig. 5). We conducted a three-way mixed-design *ANOVA* (locomotor ability [2] × flow direction [2] × dot speed [2]) and found no significant main effect or interactions regarding infants’ locomotor ability with regard to either looking time (locomotor ability: *F*(1,58) = 1.67, *p* = 0.201, *η*_*p*_^2^ = 0.03; locomotor ability × flow direction: *F*(1,58) = 1.12, *p* = 0.294, *η*_*p*_^2^ = 0.02; locomotor ability × dot-speed: *F*(1,58) = 0.09, *p* = 0.762, *η*_*p*_^2^ = 0.01; locomotor ability × flow direction x dot-speed: *F*(1,58) = 0.24, *p* = 0.624*η*_*p*_^2^ = 0.00) or latency (locomotor ability: *F*(1,58) = 1.28, *p* = 0.263, *η*_*p*_^2^ = 0.02; locomotor ability × flow direction: *F*(1,58) = 2.15, *p* = 0.148, *η*_*p*_^2^ = 0.04; locomotor ability × dot-speed: *F*(1,58) = 0.52, *p* = 0.472, *η*_*p*_^2^ = 0.01; locomotor ability × flow direction x dot speed: *F*(1,58) = 2.30, *p* = 0.135*η*_*p*_^2^ = 0.04). These results indicate that locomotor experience is not a relevant factor in the disappearance of the contraction advantage.

In view of the facts that typical optic flows during locomotion in young infants differ from those in older individuals^22^ and that locomotor experiences have no effect on the development of gaze patterns in response to the focus of radial optic flow, young infants may not rely on the focus of radial optic flow for perceiving and controlling heading direction. It is known that animals, including human beings, can use a number of visual cues other than the focus of radial flow to perceive and control heading direction. For instance, human adults can use the spatial distribution of magnifications in the change of local speed in optic flow^2^ and the location of an object in the environment^3^ to estimate and guide their locomotion paths. Honeybees reference a symmetrical structure of speed components across bilateral visual fields to maintain their flying paths^33^. In our daily locomotor behaviors, we cannot concentrate our attention entirely on the focus of radial optic flow but need to pay attention to a variety of elements in the environment (e.g., the terrain of a foot path, obstacles in a collision course, other pedestrians, etc.). Indeed, in some natural situations, even adults do not show clustered gaze patterns around the focus of radial flow during forward locomotion^29^. Analyzing the focus of radial optic flow does not represent a singular solution for perceiving and controlling heading direction; thus, applying multiple strategies related to a variety of visual cues is important with regard to increasing the reliability of our ability to monitor and control heading direction. Strategies pertaining to locomotor control, independent of the focus of radial optic flow, may be much more dominant in human infancy.

Moreover, the fact that neural^34^ and behavioral^15^ sensitivities to radial optic flow differ significantly between infancy and adulthood implies that functional links between gaze patterns in response to the focus of radial optic flow and locomotor control may also change throughout later infancy or beyond. Indeed, recent studies have shown that the development of radial optic flow sensitivity^35^ and relevant self-motion perception, such as vection^36^,^37^, continues beyond childhood and into early adolescence. It is reasonable to infer that the interaction between radial optic flow perception and control of self-motion has a protracted time course of development. Of course, as shown by previous studies^30^,^31^,^32^, some aspects of functional interactions between optic flow sensitivity and locomotion emerge in young infancy. However, the other aspect of such interactions, using the focus of radial optic flow to perceive and control heading direction, may develop in later stages of our lives, perhaps with maturation of more sophisticated locomotor actions, such as independent walking or running. Indeed, a recent empirical study^38^ using a head-mounted video camera and eye-tracking glasses revealed that optic flow experienced during locomotion is strikingly different for crawling versus for walking infants; crawlers spent more time looking down toward the ground plain and less time gazing at a target (e.g., their caregivers or a toy) representing the goal of the locomotion path than did walkers. These results suggest that differences in locomotor action can lead to different experiences of optic flow during locomotion; hence, the visual information that is required to control heading direction may be different for crawlers than it is for walkers. Future studies should investigate when the adult-like expansion bias in gaze patterns in response to the focus of radial optic flow emerges in young walkers, such as toddlers and school-aged children. Additionally, it is also important to examine the development of strategies^2^,^3^,^33^ unrelated to the detection of the focus of radial optic flow that may be used in perceiving heading direction.

## Method

### Ethics statement

This study was approved by the Ethics Committee for Psychological Research of Niigata University and was conducted according to the Declaration of Helsinki. Written informed consent was obtained from all participants (or, in case of the infants, from their parents).

### Participants

One hundred infants participated in the experiment. They were divided into five age groups; 4–6-month-olds (*N* = 20, mean age = 147.4 days, *SD* = ±27.1), 7–9-month-olds (*N* = 20, mean age = 243.4 days, *SD* = ±27.8), 10–12-month-olds (*N* = 20, mean age = 325.6 days, *SD* = ±19.0), 13–15-month-olds (*N* = 20, mean age = 436.2 days, *SD* = ±28.1), and 16–18-month-olds (*N* = 20, mean age = 520.4 days, *SD* = ±24.4). An additional eight infants also took part in the experiment but were excluded from the final results because of crying (3), inattention to visual stimuli (3), and calibration failure of the eye tracker (2). Twenty adults (*N* = 20, mean age = 20.1 years, *SD* = ±0.9) also participated in the experiment. Data from two adults were excluded from the final results because of calibration failure of the eye tracker.

### Apparatus

Experiments were conducted in an experimental booth. A 21-inch CRT monitor (Nanao, FlexScan T966, resolution = 1024 × 768 pixels, refresh rate = 60 Hz) was used to present visual stimuli. A pair of loudspeakers, placed behind the CRT monitor, made “beep” sounds at the beginning of each experimental trial to attract participants’ attention to the monitor. An eye tracker (Tobii Technology, Tobii X120) was positioned at the bottom of the CRT monitor to record participants’ gaze behaviors during the experiments. The sampling rate of the eye tracker was set at 60 Hz. The sampling rate was equivalent to or higher than that used by previous studies (e.g. refs 39, 40, 41, 42), investigating young infants’ eye movements in response to dynamic visual stimuli. Hence, we judged a sampling rate of 60 Hz to be reasonable for the purpose of testing infants’ gaze patterns in the current experiments. Stimuli presentation, recording of gaze behaviors, and data analysis were archived by Tobii Studio software (Tobii Technology) running on a personal computer (Dell, Precision T1700, CPU: Xeon E3-1240 v3 3.40 GHz, RAM: 16 GB, video card: Nvidia Quadro K600 1 GB). An LCD computer monitor was connected to the personal computer and placed outside the experimental booth to allow an experimenter to monitor the eye-tracking data and visual stimuli in real time during the experiment.

### Stimulus

All visual stimuli were presented as movies in uncompressed AVI format (resolution = 1024 × 768 pixels, frame rate = 60 frames/s, duration = 10 s). Each stimulus was composed of 500 white moving dots (luminance = 86.4 cd/m^2, size = 0.3 deg^2) presented on a dark rectangle presentation field (luminance = 8.8 cd/m^2, size = 35.3 deg × 26.4 deg). Each dot moved along a radial trajectory (either expansion or contraction) every 33.3 ms, so that the visual stimulus appeared as an animation of 30 frames/s. Dot speed was 0 deg/s at the focus of radial optic flow and increased toward the periphery. The speed of each dot (deg/s) in each animation frame was calculated by multiplying the constant (0.33 under the low-speed condition, 0.66 under the high-speed condition) by the eccentricity (in visual degrees) of the dot’s position from the focus of radial optic flow. As a result, the dot speed was zero at the focus of radial optic flow and increased linearly toward the periphery. Mean dot speeds across the whole area of a flow pattern were about 5.8 deg/s under the low-speed condition and 11.6 deg/s under the high-speed condition. Each dot had a lifetime of 10 frames (333 ms). Dots were randomly re-plotted on the presentation field at the end of their lifetime or if they moved out of the presentation field. We generated a contraction movie by playing an expansion movie in reverse order; thus, there was no difference in the visual properties of the expansion and contraction movies other than direction of dot movement. At the start of a movie, the focus of radial optic flow a flow pattern appeared at either the left or right side of the presentation field. The distance between the initial position of the focus of radial optic flow and the center of the presentation field was 9.2 deg. The focus of radial optic flow moved toward the opposite side of the presentation field during the first half of the movie (from 0 to 5 s) and then moved back to the initial position in the next half of the movie (from 5 to 10 s). Thus, the focus of radial optic flow traveled either “from right to left to right” or “from left to right to left” over the 10 seconds. The movement speed of the focus of radial optic flow was constant at 3.7 deg/s. The travel distance of the focus of radial optic flow was 18.4 deg for one-way travel (Fig. 1a).

We also used a benchmark movie to compare the ability to control eye movement among the different age groups. The movie was composed of a single white moving square (luminance = 86.4 cd/m^2, size = 4.6 deg^2) appearing on a black presentation field. The motion path of the square was identical to that of the focus of radial optic flow in an optic flow movie.

All visual stimuli used in the current study are available at “ https://nyu.databrary.org/volume/252”.

### Procedure

Infants sat on their caregivers’ lap and were held in front of the CRT monitor. Adult participants sat on a miniature chair in front of the CRT monitor without any supportive equipment, such as head and chin rest. The viewing distance was approximately 65 cm for both infants and adults. Prior to the experiment, a calibration procedure for the eye tracker was conducted by using a calibration program with five calibration points built in to the Tobii Studio software.

Before starting the experiment, the infants’ caregivers were instructed to close their eyes during the trials, so that they were naive to the stimuli identity. The adult participants were given no specific instruction. Each experimental trial started with the presentation of a fixation figure (a colorful cartoon character displayed on a white circular window [diameter = 9.2 deg]) at the center of the presentation field accompanied by beep sounds. An experimenter initiated the presentation of a flow movie when the experimenter confirmed that the participant’s gaze was on the fixation figure. Each flow movie lasted for 10 s. Each participant engaged in a total of eight experimental trials; “expansion and contraction” × “low- and high-speed conditions” × 2 repetitions (2 motion paths for the focus of radial optic flow: right/left/right or left/right/left). The order of the trials was randomized for the participants. After presentation of the eight experimental trials, each participant viewed two benchmark movies. The order of the two movies (right/left/right or left/right/left) was counterbalanced across participants (Fig. 1b).

Additionally, we conducted a behavioral observation to check the locomotor ability of infants aged 12 months or younger. The procedure of the observation was identical to that of Shirai and Imura^32^, who reported a significant developmental interaction between optic flow perception and locomotor ability in infants aged up to 12 months. After the experimental trials, each infant lay down (or sat on) a urethane play mat. An attractive toy was presented 60 cm in front of the infant. Two observers (at least one of whom was naive) checked on the infant’s behavior. If both observers agreed that the infant independently reached for the toy, the infant was regarded as a “locomotor infant.” Inter-observer agreement was 100%.

### Experimental conditions and data analyses

There were two independent factors related to the visual stimuli: flow direction (expansion and contraction) and dot speed (low and high). Thus, there were a total of four experimental conditions: expansion with low speed, expansion with high speed, contraction with low speed, and contraction with high speed.

For measurement purposes, a circular AOI was notionally defined on the screen (AOI: subtended 9.2 degrees), centered on the focus of radial optic flow (or the white square in case of a benchmark movie). During the movie presentations the AOI remained centered on the focus of radial optic flow (or the white square). Thus, the motion path of the AOI was identical to that of the focus of radial optic flow (or the white square) in each movie. Two measures regarding the participants’ gaze behaviors in response to the AOI were adopted. The first measure was the total time spent looking at the AOI in each trial, and the second measure was the latency of the participant’s first gaze at the AOI in each trial. If a participant did not gaze inside the AOI in a particular movie presentation, the latency in the presentation was regarded as 10 s (i.e., duration of the movie). Mean latency and mean looking time under each of the four experimental conditions were calculated across participants. Moreover, we also set another AOI with the aim of analyzing participants’ looking behavior with regard to the whole area of the visual stimulus, labelling it “AOIW” (AOI for the whole area of the visual stimulus). The AOIW was rectangular in shape, and its size (35.3 deg × 26.4 deg) was equivalent to that of the presentation field, so that the AOIW fully covered the whole area (including the AOI for the focus of radial optic flow) of each visual stimulus. Thus, no looking behavior relevant to the AOIW was recorded when the participant looking away entirely from the CRT monitor. The same measurements (looking time and latency) used for the circular AOI set around the focus of a radial optic flow were applied to the AOIW.

Data for the current study are available at “ https://nyu.databrary.org/volume/252”.

## Additional Information

**How to cite this article**: Shirai, N. and Imura, T. Infant-specific gaze patterns in response to radial optic flow. *Sci. Rep.* **6**, 34734; doi: 10.1038/srep34734 (2016).
